# Mechanisms of bone remodeling and therapeutic strategies in chronic apical periodontitis

**DOI:** 10.3389/fcimb.2022.908859

**Published:** 2022-07-22

**Authors:** Xutao Luo, Qianxue Wan, Lei Cheng, Ruoshi Xu

**Affiliations:** State Key Laboratory of Oral Diseases, National Clinical Research Center for Oral Diseases, Department of Cariology and Endodontics, West China Hospital of Stomatology, Sichuan University, Chengdu, China

**Keywords:** bone remodeling, chronic apical periodontitis, microorganism, signaling pathway, bone regeneration

## Abstract

Chronic periapical periodontitis (CAP) is a typical oral disease in which periodontal inflammation caused by an odontogenic infection eventually leads to bone loss. Uncontrolled infections often lead to extensive bone loss around the root tip, which ultimately leads to tooth loss. The main clinical issue in the treatment of periapical periodontitis is the repair of jawbone defects, and infection control is the first priority. However, the oral cavity is an open environment, and the distribution of microorganisms through the mouth in jawbone defects is inevitable. The subversion of host cell metabolism by oral microorganisms initiates disease. The presence of microorganisms stimulates a series of immune responses, which in turn stimulates bone healing. Given the above background, we intended to examine the paradoxes and connections between microorganisms and jaw defect repair in anticipation of new ideas for jaw defect repair. To this end, we reviewed the microbial factors, human signaling pathways, immune cells, and cytokines involved in the development of CAP, as well as concentrated growth factor (CGF) and stem cells in bone defect repair, with the aim of understanding the impact of microbial factors on host cell metabolism to inform the etiology and clinical management of CAP.

## 1 Introduction

Most periapical infections are asymptomatic and the untreated inflammation usually develops gradually and is ongoing, referred to as CAP (Paloma de Oliveira et al., 2017; Amaral et al., 2022). Infection is the most common cause of CAP, the most common of which is pulp disease, followed by apical foramen, root canal, and dentinal tubule infections. The presence of microbial factors stimulates an immune response, producing a series of pathological manifestations, mainly inflammation, and even affecting bone tissue. During treatment, infection control should be resolved first, and periapical bone remodeling should be guided on this basis. Current research focuses on CGF and stem cell research. Here we summarize the recent studies on microbes, signaling pathways, and bone remodeling to deepen the understanding of the pathogenesis of CAP and propose new treatment ideas.

## 2 Chronic apical periodontitis: a brief overview and etiology

### 2.1 Essential characteristics

Chronic apical periodontitis refers to the chronic inflammation of periapical tissues due to the long-term presence of infection and pathogenic irritants in the root canal, which is manifested by the formation of inflammation and destruction of alveolar bone. Periapical granuloma is the main type of CAP, in which apical granulation tissue is produced around the root tip of the tooth, limiting bacteria to the original infected area and activating the immune system in response to inflammatory stimuli; this process is tailored toward tissue reorganization as opposed to tissue damage. However, the loss of normal tissue is the consequence of developing tissue to fight infection (Dahlén, 2000). Notably, periapical granuloma is the early stage of CAP, and its inflammatory response is more intense than that of periapical cysts (Andrade et al., 2017). Bone loss is another clinical feature of CAP, which is caused by microbial factors and the immune defense response. However, bone loss is inexorable in apical periodontitis (AP) (Xu R et al., 2019) and repair of bone defects is difficult in AP.

### 2.2 Etiological analysis

CAP is an inflammatory disease with microbial etiology (Braz-Silva et al., 2019). Various microorganisms from the dental pulp cavity play leading roles in the development of AP (Xu R et al., 2019), and it is worth noting that bacteria account for a larger proportion of the total population than other disease-causing microorganisms, such as viruses, fungi, yeasts and protozoa (Gomes and Herrera, 2018). Typically, bacterial lipopolysaccharide (LPS) leads to AP by stimulating a local immune response (Dong et al., 2018a). When enamel or cementum is opened because of caries or trauma, oral microorganisms may enter the pulp cavity or root canal system from the crown or dentin canal and directly contact the pulp tissue or alveolar bone. However, bacteria that cause CAP are usually less toxic than those causing acute AP (Gomes and Herrera, 2018). These bacteria inhabit anatomical locations that are inaccessible to macrophages and other immune cells, such as dentin tubules, thus creating conditions for bacteria to directly damage tissue and secrete enzymes, exotoxins, and metabolic end products to regulate the immune response (Braz-Silva et al., 2019). Microleakage or the introduction of oral irritants after root canal treatment can aggravate CAP (Xu R et al., 2019). In rare cases, if the pulp has metabolic disorders or has been injured, the bacteria in the blood can be ingested by anachoresis into the pulp tissue. If the immune defense mechanism of the pulp itself cannot remove the retained bacteria, the latter can multiply in the pulp, resulting in infection.

### 2.3 Restrictions of current treatment strategies

Infection restriction and bone injury fixation are the main modalities of CAP treatment (Xu R et al., 2019). Currently, root canal therapy is the main clinical treatment for CAP. Nevertheless, the complex anatomical structure of the root canal system leads to difficulty removing the pulp cavity contents completely (Gao et al., 2018). In this case, tooth extraction or further microscopic apical surgery may be performed. Root canal microsurgery is a minimally invasive technique that not only reduces postoperative pain and dropsy, but also expedites wound healing (Floratos and Kim, 2017). According to the American Association of Endodontists, the cure rate in the microscopic treatment group can reach up to 89% at 18 months of follow-up for CAP; however, various aberrancies that may exist within the tooth still affect the therapeutic effect.

## 3 Bone remodeling of CAP

As an inflammatory condition, CAP causes an imbalance between bone resorption and reconstruction, leading to bone loss. Bone remodeling is performed by altering bone resorption and formation in chronological order. Bone resorption and formation are opposing and coupled processes of osteoblasts and osteoclasts (Yu et al., 2016), which together constitute normal bone mass. This section focuses on several factors that influence periapical bone remodeling, including microorganisms, human signaling pathways, and the immune system.

### 3.1 Microbial factors: disease promotion or alleviation

CAP is thought to be initiated by direct bacterial damage and triggers an immune response from the host, which causes the main tissue destruction process (Larsen and Fiehn, 2017). The early microbiota is simple during the pulpitis progression, and the intricacy of the root canal microbiota increases with the dominance of Gram-negative anaerobic bacteria, such as *Porphyromonas (* Larsen and Fiehn, 2017). A previous study demonstrated that among the bacteria in periapical lesions, *Fusobacteria* (4.2%), *Proteobacteria* (9.1%), *Bacteroidetes* (12.1%), *Actinobacteria* (14.0%), and *Firmicutes* (62.9%) were the main species (Korona-Glowniak et al., 2021). Moreover, 54.6% of the bacteria were strictly anaerobic, while anaerobic Gram-negative bacteria were dominant in root canals with periapical lesions (Korona-Glowniak et al., 2021). In tissues with periapical infection, bacterial abundance and diversity are significantly reduced, and the microbial balance in biofilms is disrupted (Qian et al., 2019).

Microorganisms may directly contribute to the formation and maintenance of AP, or interfere with it by influencing the host immune response. This section focuses on the former.

#### 3.1.1 LPS and LTA (lipoteichoic acid)

In general, bacterial stimulation can promote osteogenesis through synergistic action with osteogenic induction signals (Croes et al., 2019). In addition, endotoxins in the cell wall of Gram-negative bacteria, namely LPS, can cause local tissue swelling and bone absorption, mobilize the immune response, and aggravate tissue damage, and its content is positively correlated with the degree of bone damage. LPS is a TLR4 ligand (Tominari et al., 2021), which stimulates receptor activator of nuclear factor κB ligand (RANKL) expression through TLR4 signaling. In contrast to LPS, LTA is a major constituent of the cell walls of many Gram-positive bacteria. LTA induces osteoclast differentiation and bone resorption and is involved in maintaining the survival of mature osteoclasts, thereby jointly causing inflammatory alveolar bone loss (Tominari et al., 2021). LTA can also stimulate the production of prostaglandin E2 (PGE2) by upregulating genes related to PGE2 synthesis in osteoblasts and participating in subsequent inflammatory responses (Tominari et al., 2021).

#### 3.1.2 Fusobacterium nucleatum

**Figure 1:**
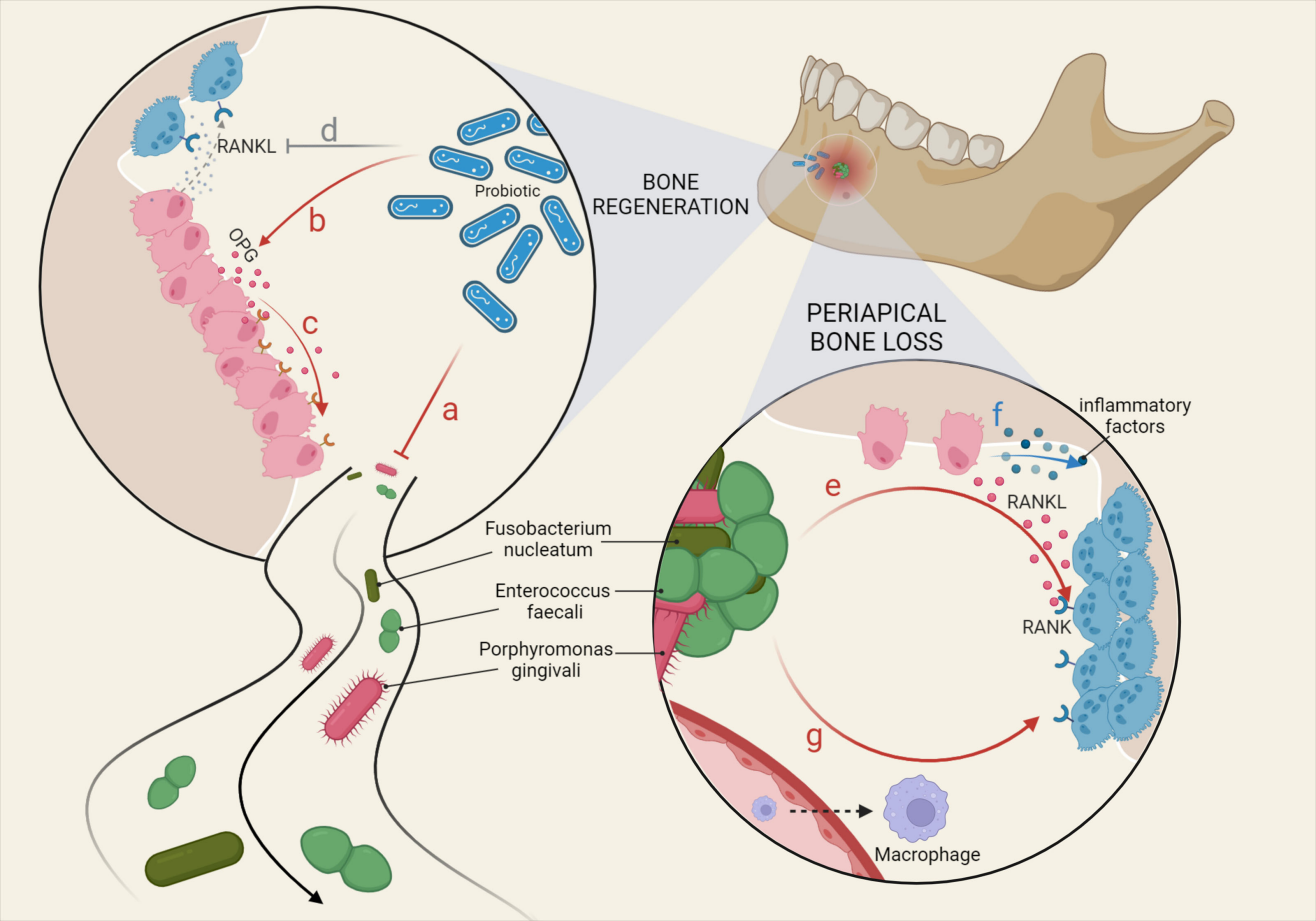
Main microbial factors affecting bone remodeling in CAP. Various oral microorganisms ultimately affect the inflammatory response and bone damage in the periapical region by influencing the physiological functions of macrophage differentiation and osteoblast and osteoclast metabolism. Bone regeneration: **(A)** Probiotics inhibit pathogenic bacteria. **(B)** Probiotics promote OPG expression by OBs. **(C)** OPG competitively inhibits the binding of RANK to RANKL. **(D)** Probiotics downregulate RANKL produced by OBs. Periapical bone loss: **(E)** Fusobacterium nucleatum and Enterococcus faecali stimulate OBs to produce RANKL, binding to RANK on OCs. **(F)** OBs are stimulated to secrete a variety of inflammatory factors, triggering greater bone loss. **(G)** LPS recruits macrophages from the blood and LTA promotes the differentiation of macrophages towards OCs.

Previous studies have shown that absorption of the alveolar bone by *Porphyromonas gingivalis*, *Campylobacter rectus*, and *Fusobacterium nucleatum* is mediated by arachidonic acid metabolites, such as prostaglandins (Gao et al., 2020). *Fusobacterium nucleatum* can act on osteoblasts, which is reflected in the reduced expression of osteogenic genes and proteins (Reis et al., 2016), inhibition of cell differentiation, formation of mineralized nodules, and increased production of pro-inflammatory factors (Gao et al., 2020) (**Figure 1**).

#### 3.1.3 Porphyromonas gingivalis

An experimental study showed that the degree of CAP caused by different pathogens varies. For the same duration of infection, CAP caused by *Porphyromonas gingivalis* was more severe in terms of bone damage and root resorption than CAP caused by *Enterococcus faecalis (* Chen S et al., 2019). Moreover, phosphoethanolamine dihydroceramide and phosphoglycerol dihydroceramide of *Porphyromonas gingivalis* can promote RANKL-mediated osteoclast formation independent of Toll-like receptor 2/4 (TLR2/4) by interacting with Myh9 (non-muscle myosin II-a) (Kanzaki et al., 2017; de Andrade et al., 2019).

#### 3.1.4 Enterococcus faecalis

Macrophages are precursors of osteoclasts. LTA of *Enterococcus faecalis* inhibits the differentiation of macrophages (RAW264.7) into osteoclasts, with no impact on the phagocytic function (Xu et al., 2018). Continuous stimulation with *Enterococcus faecalis* induces the pre-resolution polarization of macrophages into an M2 phenotype (Polak et al., 2021). In addition, *Enterococcus faecalis* can induce secretion of IL-1β and RANKL, activation of the NLRP3 inflammasome, thereby aggravating the severity of bone resorption (Reis et al., 2016; Ran et al., 2021). Xiang, Y., et al. proposed phage therapy and obtained positive results for effective inhibition of Enterococcus faecalis using this method (Xiang et al., 2022).

#### 3.1.5 Probiotics

Microorganisms not only cause CAP but have also been used in the research of new treatments. For example, some studies have explored the inhibitory effect of probiotics on pathogenic bacteria in the root canal (Kumar et al., 2021), which may be used to identify pathogenic bacteria in the root canal for the purposes of treatment (Bohora and Kokate, 2017). The results of a comparative clinical trial showed that treatment with a probiotic product completely prevented the regeneration of biofilms formed by Enterococcus faecalis compared to low concentrations of sodium hypochlorite solution (Safadi et al., 2022). Indeed, probiotics also inhibit bone resorption by increasing osteoprotectin (OPG) and decreasing RANKL (Kumar et al., 2021) (**Figure 1**).

### 3.2 RANKL-RANK-OPG system

RANKL is the ligand required for osteoclast formation, whereas RANK and OPG are the receptor and decoy receptor of RANKL, respectively. The RANKL-RANK-OPG system plays an important role in mandibular bone remodeling (Aguirre et al., 2021).

#### 3.2.1 RANKL

RANKL is a pivotal cytokine in bone resorption, and facilitates bone dissolution while continuously promoting periapical inflammation (Francisconi et al., 2018). Previous studies have shown that LPS (Dong et al., 2018b), IL-6,TGF-β, parathyroid hormone -related protein (PTHrP) (Yasuda, 2021), IGF-1 (Huang et al., 2018; Yasuda, 2021), and other inflammatory factors facilitate RANKL expression. During osteoclast differentiation, activated T nuclear factor (NFATc1) is a major transcription factor, which can be activated by RANKL binding to its receptor RANK to promote osteoclast differentiation (Canalis et al., 2021). Additionally, TNF receptor-associated factor 6 (TRAF6) in osteoclast precursors is necessary to mediate RANKL-induced NF-κB activation and osteoclast formation and activation. Studies have shown that RANKL contributes to periapical granulomas and cysts (Santos et al., 2017). Chronic infiltration is strongly associated with high expression of RANKL, which represents increased osteolytic activity (Santos et al., 2017). A reverse RANKL signaling was also detected, which served to restore alveolar bone loss in mice (Ozaki et al., 2017). Therefore, the RANKL reversal signaling modulator may be a promising candidate drug action site for the treatment of AP bone loss (Ozaki et al., 2017). The experimental results of a rat AP model showed that the SPHK1-S1PR1-RANKL axis regulates inflammatory bone imbalance (Xiao et al., 2018). The activity of SPHK1 was significantly increased in macrophages stimulated by LPS, which activated S1PR1 in Bone marrow Mesenchymal Stem Cells (BMSCs), resulting in increased expression of RANKL in BMSCs. Additionally, the RANKL/RANK/OPG system plays an important role in the regulation of the immune system (Kimura et al., 2020), principally *via* regulation of the phenotype and function of dendritic cells by RANKL (Lin et al., 2016). A previous study reported that inflammatory damage from the activate to inactive state in CAP may occur as a result of RANKL immunoregulatory feedback, and dendritic cells seem to be the underlying factor determining whether this transition is possible (Cavalla et al., 2021).

#### 3.2.2 OPG

OPG, the inducible receptor of RANKL, is mainly produced by osteoblasts and has a higher affinity than RANK (Canalis et al., 2021). It has been reported that during CAP, IL-17, IFN-ϒ, and higher concentrations of IL-33 upregulate RANKL production (Duka et al., 2019). The expression of OPG is upregulated by IL-10 and low concentrations of IL-33, which inhibits bone dissolution (Duka et al., 2019). However, another study reported contradictory results, indicating that IL-33 was highly expressed in CAP and was negatively correlated with the expression of RANKL but positively correlated with the expression of OPG (Gegen et al., 2019). One possible explanation is that even though IL-33 expression is higher than normal in periapical lesions, its high expression remains insufficient to promote RANKL and inhibit OPG generation. However, this conjecture requires further experimental confirmation.

#### 3.2.3 RANKL/OPG ratio

The RANKL/OPG ratio is widely used as a reflective indicator of bone formation and absorption. Evidence has shown that the RANKL/OPG ratio is a key determinant of the progression or stability of periapical lesions (Cavalla et al., 2021). An increase in this ratio indicated increased bone loss. One experimental study showed that the RANKL/OPG ratio in the periapical granuloma group was significantly higher than that in the periapical cyst group, suggesting significantly more bone absorption in periapical granuloma (Takahama et al., 2018). This is associated with an increased inflammatory response and pathological microbial activity in periapical granulomas (Andrade et al., 2017). The RANKL/OPG ratio is mainly used to distinguish healthy from diseased periapical, but it lacks the ability to distinguish symptomatic from asymptomatic periapical. However, the detection of the TRAP5 level meets these requirements (Salinas-Muñoz et al., 2017).

RANKL may be involved in the activation of the inflammatory state of CAP through a feedback mechanism of the immune system. Moreover, its stimulatory effect on osteoclast maturation and differentiation can be inhibited by OPG. In addition, osteopontin (OPN) has also been shown to affect the binding of RANKL to OPG *via* the NK-kB pathway to promote osteolysis, which may be related to N-glycosylation during OPN formation (Dong et al., 2022).

### 3.3 Signaling pathways

#### 3.3.1 Notch signaling

The Notch signaling pathway is a highly evolutionarily conserved ligand receptor signaling pathway that plays a crucial role in cell fate determination during cell survival, homeostasis, proliferation, differentiation, and development (Lee et al., 2021). Notch1 inhibits osteoclast formation, whereas Notch2, through both direct and indirect mechanisms, promotes osteoclast differentiation and function (Yu and Canalis, 2020). In periapical disease, Notch2 affects the clinical attachment level and is considered to be involved in alveolar bone loss. Downregulation of Notch1 may occur in severe osteolysis following RANKL activation (Djinic Krasavcevic et al., 2021). Notch3 also induces RANKL expression in osteoblasts and osteocytes (Yu and Canalis, 2020). However, Notch4 is less expressed in bone cells (Yu and Canalis, 2020). Another study suggested that the Notch signaling pathway may participate in alveolar bone resorption in Epstein-Barr virus infected periapical lesions (Jakovljevic et al., 2020). According to a recent study, basic levels of Notch3 are essential for skeletal bone mass balance, whereas activated Notch3 in osteoblasts and osteocytes suppresses osteoclast formation and bone resorption in cancellous bone (Canalis et al., 2021). Additionally, it has been hypothesized that the Notch signaling pathway increases Notch receptors on the surface of immune cells and stimulates the transposition of the Notch receptor intracellular domain (NICD) to the nucleus (Jakovljevic et al., 2019).

#### 3.3.2 Wnt/β-catenin signaling

Wnt signaling is a vital regulatory pathway in osteoblast differentiation (Zhang and Zhang, 2017). The regulation of Wnt/β-catenin signaling pathway inhibitors intensifies periapical lesions (Naruse et al., 2021). The effect of Wnt3a treatment on osteogenic function is temporally dichotomous (Tang et al., 2014). Interactions between signaling pathways may also exist. Upregulation of NF-κB signaling in LPS-induced inflammation can be inhibited by inhibition of the Wnt3a/β-catenin signaling pathway (Guan et al., 2021). It is speculated that simultaneous blocking of these two signaling pathways can significantly inhibit the development of AP. In addition, the secretion of crimp-related protein I (sFRP1) is involved in the osteogenic differentiation of HPLCs under inflammatory conditions by antagonizing Wnt (Yang F et al., 2021). Both the promotion of bone repair by berberine and the role of Dickkopf-1 in mediating bone damage are associated with the regulation of the Wnt/β-catenin signaling pathway (Tan et al., 2018; Cui et al., 2020).

#### 3.3.3 TGF-β signaling

In mice with conditional deletion of TGFβ R2 (a major receptor for TGFβ signaling) from early mesenchymal progenitor cells, alveolar bone mass and density were significantly reduced during early development and the periodontal ligament was severely damaged (Xu et al., 2021). Bone morphogenetic proteins (BMPs) belong to the TGF-β family, which is a group of highly conserved and structurally similar functional proteins. BMPs are a research hotspot in bone regeneration technology. Among the members of the BMP family, BMP9, BMP7, BMP6, BMP4, and BMP2 are known to induce osteogenesis (May et al., 2019). Mineralized nanofiber fragments and BMP-2 mimic peptides have the potential to induce alveolar bone regeneration (Boda et al., 2019). As biocompatible scaffolds, carbonate apatite scaffolds can upregulate the expression of BMP7 and BMP2, decrease the expression of MMP8, and support the proliferation and differentiation of mesenchymal stem cells (Prahasanti et al., 2020). The presence of BMP-2 and mesenchymal stem cells can accelerate bone formation (Stutz et al., 2020). Another study showed that the combination of BMP-2/7 non-viral vectors with *in vivo* electroporation has potential as a non-surgical treatment option for alveolar bone regeneration (Kawai et al., 2018).

#### 3.3.4 5-Lipoxygenase signaling

In a mouse model of AP, activating the 5-LO pathway stimulated the synthesis of inflammatory mediators and inhibited osteoclast formation (Paula-Silva et al., 2020). Another study showed that 5-LO inhibitors can significantly reduce the number of inflammatory cells and osteoclasts, and bone resorption by affecting leukotriene B4 (LTB4) levels (Lopes et al., 2017). In the early development of the disease, osteoclasts are reduced because of the inhibition of 5-LO (Paula-Silva et al., 2016). However, when 5-LO inhibition is longstanding, it is difficult to block the synthesis of catabolic mediators, so the recruitment of inflammatory cells and bone absorption are not reduced (Paula-Silva et al., 2016). Another study examined 5-LO in periapical mice from a holistic perspective. Compared to the control group, 5-LO deficient mice had a greater periapical bone damage area, and significantly increased levels of inflammatory factors and osteoclast-forming factors, including TNF-α, IL-1β, and RANKL, while OPG was decreased, suggesting that the innate immune system of mice with 5-LO deficiency was damaged and periapical inflammation was aggravated (Wu et al., 2018).

#### 3.3.5 TLR signaling

Toll-like receptors (TLRs) are pathogen recognition receptors encoded by a variety of germ lines expressed by antigen-presenting cells, which induce their maturation, lead to gene transcription, and produce a variety of pro-inflammatory and anti-inflammatory cytokines (Desai et al., 2011). TLRs play important roles in the initial recognition of periapical pathogens. TLR4 has pro-inflammatory properties and can be activated by LPS or endogenous damage-associated molecular patterns (DAMPs), promoting an increase in NF-kB and pro-inflammatory factors (Deng S et al., 2020; Ciesielska et al., 2021). Interestingly, the activity of LPS in *Porphyromonas gingivalis* is mediated exclusively by TLR4 (Nativel et al., 2017). In periapical granuloma, TLR2 is highly expressed in lymphocytes and plasma cells, but with reduced expression in dendritic cells, suggesting that the former two cell types play a more important role in the disease than the latter (Chen et al., 2018). Another study showed that in apical lesions, TLR2 and TLR4 are overexpressed and associated with collagenolytic MMPs (Fernández et al., 2019). Experiments demonstrated that TLR signaling related genes in TLR4, NF-κB, TNF, CD14, MyD88, and IL-6 were gradually upregulated during the progression of CAP and were highly expressed during the formation of bone destruction (Hasegawa et al., 2021).

In future therapeutic strategies at the molecular level of CAP, further investigation of BMP can lead to its clinical translation by inducing the transmission of Notch1, Notch3, and 5-LO signaling pathways, inhibiting Notch2, and downregulating the expression of the Wnt/β-catenin and NF-kB signaling pathways to achieve the desired effect.

### 3.4 Production and destructive effects of reactive oxygen species at the site of inflammation

Oxidative stress is commonly seen in a variety of inflammatory diseases. The hypoxic environment of periapical inflammation can drive the progression of periapical inflammation by inducing ROS production as well as apoptosis of osteoblasts (Lai et al., 2018). ROS modulates cell signaling to increase the production of pro-inflammatory mediators and MMP and may exacerbate alveolar bone resorption by stimulating RANKL-mediated osteoclast differentiation and inhibiting osteoblast differentiation (Hernández-Ríos et al., 2017; Georgiou et al., 2021). SIRT5 can mitigate hypoxia-induced osteoblast apoptosis by a mechanism that may be related to Mitochondria (Yang CN et al., 2021).

### 3.5 Epigenetics

#### 3.5.1 DNA methylation

DNA methylation, which can modify gene expression patterns without altering the DNA sequence, refers to the covalent bonding of methyl to the cytosine 5’ carbon position of genomic CpG dinucleotides in the presence of DNA methylation transferase. Campos, K., et al. found that all periapical lesion samples exhibited partial or full IFNG gene and that the partially methylated samples showed increased expression of the corresponding mRNA (Campos et al., 2013). The team also found that the FOXP3 gene promoter showed high methylation levels in both periapical granulomas and apical cysts, with corresponding mRNA expression downregulated and associated with downregulation of IL-10 and TGF-β (Campos et al., 2015). Wichnieski, C., et al. showed that the differential methylation profiles of FADD, CXCL3, IL-12B and IL-4R contribute to disease-related potential contribution of altered gene expression (Wichnieski et al., 2019). Bordagaray, M.J., et al. found that TLR2 upregulation was associated with methylation of a single CpG site in the TLR2 gene promoter (Bordagaray et al., 2022), while Fernández, A., et al. showed that TLR2 gene promoter hypomethylation was associated with increased transcriptional activity of pro-inflammatory cytokines and angiogenic markers in periapical inflammation (Fernández et al., 2020). Demethylation of the CpG site of the TLR9 promoter, as well as the DNA methylation status within the gene, has also been shown to be associated with lesions in periapical inflammation (Fernández et al., 2022). Both methylation and demethylation of DNA are associated with bone conversion. The methylation status determines the propensity of bone marrow-derived MSCs to differentiate towards osteoblasts, which may be associated with hypomethylation of the promoter regions of the osteogenic genes RUNX2 and OCN, and higher expression of the corresponding mRNAs. In addition, methylation regulation of the Wnt/β-catenin signalling pathway and the RANKL/RANK/OPG system also plays an important role in bone regeneration (Oton-Gonzalez et al., 2022). The studies related to DNA methylation in periapical inflammation are currently inadequate, and the elucidation of the relevant mechanisms depends on further studies.

#### 3.5.2 miRNA

The contribution of miRNAs to bone remodeling in CAP has also been extensively studied (**Figure 1**). Several miRNAs have been identified in AP (Shen et al., 2021), but only a few have been thoroughly studied. Among them, mir-10A-5p has the highest expression level, and its overexpression can downregulate TNF-α mRNA levels and upregulate IL-10 (Shen et al., 2021). mir-155 inhibits SEMA3A in the progression of AP (Yue et al., 2016). mir-335-5p positively contributes to the inflammation of HPDLF, and its targets are RANKL and uPAR. Regardless of inflammation, mir-335-5p can promote the expression of RANKL in HPDLFs, while uPAR is suppressed by mir-335-5p, which can be alleviated by LPS stimulation (Yue et al., 2017). Moreover, mir-335-5p promotes osteogenic differentiation in mice by downregulating the Wnt antagonist, Dickkopf-1 (DKK1) (Zhang et al., 2017). mir-200a takes part in the migration of BMSCs induced by *Enterococcus faecalis* products through the FOXJ1/NFκB/MMPs axis, thus regulating bone injury repair (Li et al., 2020). mir-181b-5p negatively regulates THF-α-induced inflammation by targeting IL-6 in cementoblasts and modulating the NF-κB signaling pathway while promoting osteoblast apoptosis (Wang et al., 2019). Another study inferred that mechanical stress-induced exosomes facilitated the proliferation of HPLSCs through the mir-181-5p/PTEN/AKT signaling pathway and facilitated their osteogenic differentiation through BMP2/Runx2 (Lv et al., 2020). An experiment showed that mir-146a is also LPS-induced and is a negative mediator of inflammation that downregulates the expression of TNF-α, IL-1β, and IL-6 (Lina et al., 2019). Meanwhile, it was demonstrated that Hey2 is the target gene of mir-146a, which together form a regulatory loop and negatively regulate each other (Lina et al., 2019). Furthermore, exosomal mir-1260b inhibits osteoclast formation *via* the WNT5a-mediated RANKL pathway (Nakao et al., 2021).

### 3.6 Main immune cells mediate the major tissue destruction process

#### 3.6.1 T Cells

The activity of osteoclasts in CAP is influenced by T-cell regulation (Wang et al., 2021). The functions of the T lymphocyte family, including Th1, Th2, Th17, and regulatory T (Treg) cells, have been extensively described. Recent studies have focused on Th17 and Treg cells, which have opposite roles in the immune response in periapical lesions (Toledo et al., 2019). Treg and Th17 cells are thought to be important junctions between the immune system and bone (Zhang et al., 2021). According to a recent review, IL-17 and TNF-α secreted by Th17 cells promote RANKL expression and osteoclast differentiation, while TGF-β and IL-10 secreted by Treg cells inhibit this process (Wei et al., 2021). Th17 cells also stimulate the colony of neutrophils, triggering an inflammatory response to stimulate osteoclast activity, whereas neutrophil clearance of infection inhibits osteoclast activity (Wei et al., 2021). In addition, IL-1β, IL-6, IL-21, IL-23, and in periapical inflammatory environment can stimulate the upregulation of STAT3 and NFAT in Th17 cells, further increase the levels of IL-22, IL-21, IL-17F, IL-17A, and pathologically up-regulate the expressions of IFN-γ and GM-CSF (Hasiakos et al., 2021).

#### 3.6.2 B Cells and plasma cells

Plasma cells play a more important role in tissue repair than CAP development (Weber et al., 2019). The intensity of the plasma cell response is determined by its number, with plasma cells being more numerous and responsive in older, neglected granulomas than in more recent ones (Bănică et al., 2018). A mouse model suggested that antigen-activated B cells significantly increase RANKL expression and promote osteoclast generation (Settem et al., 2021). Switched memory B cells produce more RANKL and increase Th1 and Th17 cell proliferation to stimulate osteoclast and alveolar bone loss (Han et al., 2018). In contrast, in periodontitis, B cells may play an active role in suppressing inflammation and osteolysis (Zeng et al., 2021). However, the mechanism underlying the roles of B cells in periapical periodontitis has rarely been described, and the mechanism in periodontitis can only be used as a reference. Therefore, further experiments are required to study B cells in periapical periodontitis.

#### 3.6.3 Macrophages

Macrophages are involved in both innate and adaptive immunity, and modulate the immune response to host inflammation. One report has shown that AP antigen presenting cells, such as macrophages, may have a greater role than T cells in the pathogenesis of AP (Weber et al., 2019). Macrophages in periapical lesions show polarization switches towards M1 (Veloso et al., 2020), and this polarization process is triggered by pathogen-associated antigens. Macrophage polarization may be related to the development and progression of periodontitis injury, including alveolar bone loss (Weber et al., 2018). Th1 T cells and phagocytes promote bone resorption by upregulating the expression and secretion of inflammatory factors and RANKL (Hienz et al., 2015). The development of exacerbated inflammation in AP is likely to be distinctly influenced by the macrophage migration inhibitory factors -794 CATT5-8 and -173G>C (Freer-Rojas et al., 2020). One study showed that the expression of PD-L1 was more significant in macrophages at the focal site of CAP than in the control group (Delgado et al., 2019). PD-L1 binds to PD-1 on T cells to inhibit immune function, which may be related to the persistence of bacteria in CAP and chronic pulp infection.

#### 3.6.4 Mast cells

Mast cells (MCs) also contribute to bone damage in periapical periodontitis (Sheethal et al., 2018). MCs can promote bone destruction by secreting the classic pro-inflammatory factor TNF-α. In addition, the number of MCs with MMP-8 and MMP-13 double-positive in AP was significantly increased (Wang G et al., 2018). In contrast, TGF-β expressed by MCs seems to neutralize IL-1, TNF-α, and other pro-inflammatory factors and inhibit macrophage activity (Tang et al., 2017). From an immune perspective, bone loss due to inflammatory bone imbalance has positive roles because bone resorption creates space for immune cells to infiltrate, thus forming a barrier against infection (Holland et al., 2017).

### 3.7 Relative cytokines

Cytokines are diverse and play complex roles in inflammatory responses. In this section, we discuss colony stimulating factor (CSF), tumor necrosis factor (TNF), interleukin (IL), and interferon (IFN) (**Table 1**).

**Table 1:** Roles of cytokines and miRNA.

Names	Functions			References
**IL**				
IL-4	prevents bone damage			(Freire et al., 2021)
IL-12	(1)increases MMP-1, MMP-3 and MMP-13; (2)inhibits MMP-2 and MMP-9			(Ma et al., 2017; Miao et al., 2017)
IL-17	(1)up-regulates RANKL through the JAK2-STAT3 pathway;(2)promotes osteoclast differentiation			(Wang Z et al., 2018; Xiong et al., 2019; Wei et al., 2021)
IL-17a	(1)recruiting neutrophils			(Ferreira et al., 2016)
IL-10	(1)inhibits RANKL expression;(2)inhibits osteoclast differentiation;(3)up-regulates OPG			(Duka et al., 2019; Wei et al., 2021)
IL-22	bone destruction			(de Oliveira et al., 2015)
IL-34	binds to RANKL and stimulates osteoclast formation			(Ma et al., 2016)
IL-33	(1)higher concentration of IL-33 up-regulates RANKL;(2)lower levels of IL-33 up-regulates OPG			(Duka et al., 2019)
IL-1β	bone resorption(osteoclast formation)	(1)increases the levels of IL-17A, IL-17F, IL-21, and IL-22;(2)pathologically up-regulates the expressions of IFN-γ and GM-CSF		(Ma et al., 2017; Veloso et al., 2020; Zhang Z et al., 2020; Hasiakos et al., 2021)
IL-6	(1)promotes the expression of RANKL;(2)promote functional osteoclast differentiation			
IL-23	stimulates osteoclast formation in LPS-induced PDL cells			
IL-21	::			
**IFN**				
IFN-γ	(1)up-regulates RANKL;(2)attenuates the promoting effect of IL-17 on gene expression of Alp, Runx2, Osteocalcin, OPG and RANKL;(3)promotes early differentiation of osteoblasts, but negatively modulates osteoblast calcification			(Wang Z et al., 2018; Wobma et al., 2018; Duka et al., 2019)
IFN-α	anti-osteoblast cytokines			(Amarasekara et al., 2021)
IFN-β	strong inhibitor of osteoclast formation			(Zheng et al., 2017; Amarasekara et al., 2021)
IFN-λ1	inhibits osteoclast formation			(Chen et al., 2020)
***CSF***				
MCSF	facilitates the proliferation process of osteoclast precursor cells and helps maintain the survival of osteoclasts		impacts macrophage	(Anesi et al., 2019)
GCSF	(1)increases the expression of inflammatory factors such as CXC chemokines, interleukins (IL-1β, IL-6) and MMP-9;(2)up-regulates the ratio of RANKL/OPG and the number of osteoclasts			(Zhang Z et al., 2020)
**TNF**				
TNF-α	(1)activates osteoclasts and inhibits collagen synthesis;(2)directly affects osteoblast expression of osteolytic cytokines through NF-κB signaling pathway;(3)up-regulates M-CSF to indirectly affects osteoclast formation and activity;(4)up-regulates the release of prostaglandin E2 from osteoblasts;(5)alleviates bone loss *via* up-regulating exosome CD73 expression and inducing polarization of M2 macrophages;(6)promotes RANKL expression and promotes osteoclast differentiation		promotes functional osteoclast differentia -tion	(Yu et al., 2016; Wang et al., 2017; Canalis et al., 2021; Wei et al., 2021)
TNF-β	activates osteoclasts and inhibits collagen synthesis	
**miRNA**				
mir-10A-5p	down-regulates TNF-A mRNA levels and up-regulates IL-10			(Shen et al., 2021)
mir-155	inhibits SEMA3A			(Yue et al., 2016)
mir-335-5p	(1)promotes the expression of RANKL in HPDLFs;(2)inhibits uPAR;(3)promotes osteogenic differentiation in mice			(Yue et al., 2017; Zhang et al., 2017)
mir-200a	takes parts in migration of bone marrow mesenchymal stem cells			(Li et al., 2020)
mir-181b-5p	(1)promotes osteoblast apoptosis;(2)modulates the NF-κB signaling pathway			(Wang et al., 2019; Lv et al., 2020)
mir-146a	negatively regulates the expression of IL-6, IL-1β and TNF-α			(Lina et al., 2019)
mir-1260b	inhibits osteoclast formation through the WNT5a-mediated RANKL pathway			(Nakao et al., 2021)

#### 3.7.1 CSF

The proliferation and survival of osteoclasts before differentiation are mainly regulated by MCSF (Anesi et al., 2019). An *in vitro* assay showed that M-CSF anti-c-fms antibodies did not alter the expression of RANKL and OPG during osteoblast and osteoclast differentiation but directly inhibited osteoclast precursors to osteoclast formation (Nara et al., 2020). LPS can induce the production of TNF-α in osteoblasts in CAP induced by LPS from *Porphyromonas* in dental pulp. The expression of M-CSF in apical cysts was significantly higher than that in apical granulomas, indicating increased osteoclast activation and continuous bone resorption in periapical cysts (Weber et al., 2019). In addition, RANKL was not significantly upregulated in periapical cysts compared to in granulomas (Weber et al., 2019). GCSF stimulates bone tissue injury by increasing the expression of inflammatory factors such as CXC chemokines, interleukins (IL-1β, IL-6), and MMP-9, and increasing the ratio of RANKL/OPG and the number of osteoclasts (Zhang Z et al., 2020).

#### 3.7.2 TNF

TNF-α and TNF-β can activate osteoclasts and inhibit collagen synthesis, and are mainly produced by macrophages and activated lymphocytes, respectively. TNF-α directly affects osteoblast expression of osteolytic cytokines, such as M-CSF, through the NF-κB signaling pathway, and indirectly affects osteoclast formation and activity through the action of M-CSF (Yu et al., 2016). TNF-α can also up-regulate the release of prostaglandin E2 from fibroblasts and osteoblasts (Canalis et al., 2021), and contributes to the alleviation of bone loss by increasing exosome CD73 expression and inducing the polarization of M2 macrophages. In addition, TNF-α inhibits the role of BMP9-induced osteoblast stem cells in inflammatory processes in mouse apical papilla osteogenesis (Wang et al., 2017). Nonetheless, the role of TNF in promoting osteoclast precursor differentiation is inhibited by multiple mechanisms, such as the RBP-J-mediated regulatory network, which has been reviewed and described in detail (Zhao, 2020). This may explain why TNF-mediated osteoclast formation is much weaker than RANKL-mediated osteoclast formation. Moreover, at the molecular level, the TNF-α-induced inflammatory response in odontoblast cells can be down-regulated by mir-181b-5p (Wang et al., 2019).

#### 3.7.3 IL

Among the pro-inflammatory cytokines in the periapical granulation tissue, Interleukin-1 β (IL-1β) is the most common. TLR and inflammasome activation contribute to the regulation of synthesis and secretion of IL-1β (Ran et al., 2021). With the progression of periapical inflammation, the expression levels of IL-1α and IL-1β in deciduous teeth increase in periapical granulomas, which may be a cause of inflammation (Yang et al., 2018). The main role of IL-4 in AP is to prevent bone damage and inhibit the development of inflammation and the resulting injury (Freire et al., 2021). IL-6 and IL-23 (Veloso et al., 2020) are associated with the severity of apical lesions. IL-23 stimulates osteoclast formation in LPS-induced PDL cells (Ma et al., 2017). IL-12 may regulate the expression of MMP in hPDLFs through the NF-κB signaling pathway, in which the expression levels of MMP-13, MMP-3, and MMP-1 are increased, while the expression levels of MMP-9 and MMP-2 are inhibited (Ma et al., 2017; Miao et al., 2017). IL-17 also contributes to bone resorption in periapical inflammation (Xiong et al., 2019), possibly through RANKL upregulation (Duka et al., 2019). A previous study confirmed that IL-17 can directly promote RANKL expression through the JAK2-STAT3 pathway (Wang Z et al., 2018; Xiong et al., 2019). In periapical abscesses, IL-17a is highly expressed and responsible for the initiation and subsequent progression of inflammation, as well as recruitment of neutrophils to the site of infection (Ferreira et al., 2016). In a mouse model, IL-22 deletion resulted in a smaller periapical lesion area and lower bone destruction, suggesting the promoting role of IL-22 in CAP (de Oliveira et al., 2015). IL-34 may bind to RANKL and stimulate osteoclast formation in CAP (Ma et al., 2016).

#### 3.7.4 IFN

Interferon inhibits osteoclast formation in many chronic inflammatory conditions and is associated with bone loss (Place et al., 2021). IFN-γ upregulates RANKL production (Duka et al., 2019). In mesenchymal stem cell therapy, IFN-γ promotes the expression of anti-pathogenic proteins and induces the action of mesenchymal stem cells while promoting their own survival, resulting in the inhibition of inflammation and fibrosis (Wobma et al., 2018). IFN-γ may have a promoting effect of IL-17 on the expression of OPG, Runx2, Alp, and RANKL (Wang Z et al., 2018). Furthermore, IFN-γ promotes the early differentiation of osteoblasts but negatively modulates osteoblast calcification (Wang Z et al., 2018). IFN-γ is considered an osteoblast cytokine, while both IFN-α and IFN-β are anti-osteoblast cytokines (Amarasekara et al., 2021). IFN-β is a strong inhibitor of osteoclast formation, as evidenced in the following two cases. One of these is the farnesoid X receptor, whose deletion enhances osteoclast formation by downregulating IFN-β expression *via* RANKL and disrupting the downstream JAK3-STAT1 signaling pathway (Zheng et al., 2017). The other is Def6, which has been identified as a regulator of bone remodeling and a key upstream regulator of IFN-β expression (Deng Z et al., 2020). IFN-λ1 prevents LPS-induced inflammatory bone damage by suppressing osteoclast formation and bone resorption (Chen et al., 2020).

### 3.8 Inflammasome

NLRP12 reduces inflammation and osteoclasts by negatively regulating the NF-κB pathway (Taira et al., 2019). NLRP6 suppresses the production of inflammatory promoters such as IL-6 and TNF-α in HPLCs by inhibiting the NF-κB and ERK signaling pathways (Lu et al., 2019). NLRP3 is also associated with AP progression and its ubiquitination is critical for ATP-induced IL-1ß secretion and the alleviation of LPS by TRIM31 (Wu et al., 2020). Experimental results suggest that NLRP3 may be a promising target for the prevention and treatment of periodontal inflammation induced by *Enterococcus faecalis (* Ran et al., 2021).

### 3.9 Matrix metalloproteinases

The MMP family is extensively involved in tissue destruction during inflammation, including alveolar bone absorption during periapical inflammation. In inflammation, the expression of MMP-9, MMP-7, and MMP-2 is increased (Letra et al., 2013), and the expression levels of MMP-13 and MMP-8 in MCs are also promoted (Wang G et al., 2018). MMP-1 is a key enzyme in initial bone resorption during periapical injury (Jain and Bahuguna, 2015), while MMP-2 is also involved in bone resorption (Yu et al., 2018). MMP-8 and MMP-13 are related to the pathological response to inflammation, while MMP-9 seems to suppress inflammation (Zhang H et al., 2020), which can reduce the expression of various inflammatory factors and osteoclast factors induced by LPS stimulation, while also upregulating the expression of OPG and osteocalcin (OCN). The upregulation of MMP-9 expression levels may be related to the inflammatory state that deregulates the methylation of DNA in the promoter region (Ahmed et al., 2021). A clinical study has shown that the use of calcium hydroxide as an intracanal medication during root canal treatment can lead to lower levels of MMP-9 synthesis (Paula-Silva et al., 2021).

## 4 Advances in alveolar bone regeneration after CAP

Most endodontic treatments are successful, but in a small percentage of cases of periapical inflammation there is persistence of symptoms or recurrence (Sureshbabu et al., 2020). Periapical surgery is the treatment of choice in such cases. Bone regeneration at the newly formed wound after endodontic surgery is an important step in periapical restoration (Montero-Miralles et al., 2021). The application of CGF or stem cells is an effective means to ensure a rapid and successful recovery of the diseased periapical area after surgery (Keranmu et al., 2021).

### 4.1 Traditional method: periosteum and bone powder

The traditional method of bone regeneration for AP involves the use of the periosteum or bone powder. The periosteum consists mainly of heterogeneous cells, extracellular matrix scaffolds, blood vessels, and nerves. The periosteum mainly plays an osteogenic role through periosteum stem cells (Debnath et al., 2018), whereas the extracellular matrix of the periosteum plays a synergistic role in osteogenesis (Sun et al., 2019). In addition, the blood supply to the periosteum is rich (Lou et al., 2021), which is of great significance for the formation of new bone. Bone powder is widely used to guide bone regeneration. Bio-Oss is a carbonate apatite crystal extracted from bovine bones, with a similar structure to that of human bone, with good biocompatibility and the ability to induce bone regeneration; thus, it is widely used in clinical practice (Wang et al., 2020).

### 4.2 Concentrated growth factors

An increasing number of clinical studies have shown that CGFs can achieve rapid repair and regeneration of periapical lesions, and using CGF as a substitute for the periosteum and bone powder is considered a better strategy for periapical bone regeneration (Sureshbabu et al., 2020). CGF, a new generation of platelet concentrate with more abundant and thicker growth factor content than platelet -rich fibrin (PRF), is a highly concentrated collection of growth factors (Sureshbabu et al., 2020). CGF is mainly composed of concentrated platelets, white blood cells, cytokines, and fibrin with a reticular structure, and is prepared with relation to the coagulation of blood. CGF contains TGF-1, platelet-derived growth factor (PDGF), vascular endothelial growth factor (VEGF), epidermal growth factor (EGF), and insulin-like growth factor-1 (IGF-1), among others (Keranmu et al., 2021; Vaid et al., 2021). The scaffold network structure of CGF slows the release of the growth factors. As VEGF has a short half-life, the slow-release effect of CGF can alleviate the disadvantage of VEGF as a free protein, which necessitates a large amount and carries a high cost (Stanca et al., 2021). TGF-β1 is the most abundantly released growth factor in CGF (Stanca et al., 2021). According to one study, the longest release time of the factors in CGF was 28 days (Stanca et al., 2021). The authors also found that BMP-2 was the least abundant and released factor in CGF.

The growth factors and cytokines mentioned above stimulate osteoblast activity (Zoltowska et al., 2021). TGF-β1 stimulates the expression of bone morphogenetic proteins and inhibits matrix metalloproteinases (MMPs) and other enzymes to stimulate osteoblast differentiation, while VEGF promotes angiogenesis, which occurs before bone formation, and is key to bone regeneration. A promising effect of sticky bone on long-term regeneration has also been anticipated (Zoltowska et al., 2021). An evaluation study showed that CGF can perform better as a scaffold when used in combination with Bio-Oss (Xu Y et al., 2019).

### 4.3 Mesenchymal stem cells

**Figure 2:**
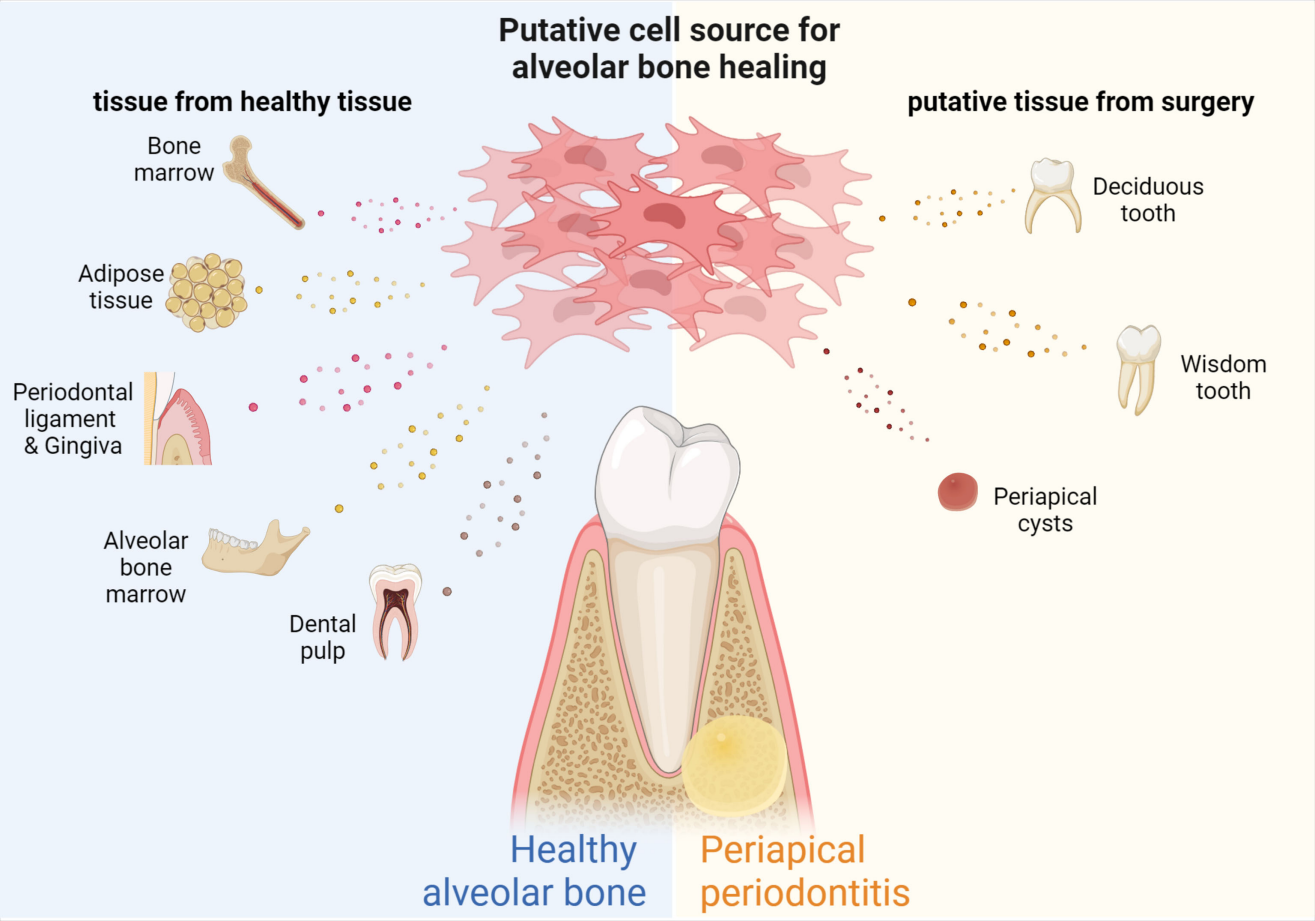
Source of MSCs for bone regeneration in periapical periodontitis. MSCs with different properties can be extracted from different tissues of the human body, including SCAPs and DPSCs from teeth, PDLSCs and GMSCs from periodontal tissue, BMSCs from bone marrow, ABMSCs and MSCs from alveolar bone, and ADSCs from adipose tissue, human milk teeth, apical cysts, while wisdom teeth can also be used as biomaterials for obtaining MSCs.

In the natural pathology of CAP, the tissue at the site of inflammation has the ability to recruit stem cells (Farias et al., 2022), but endogenous stem cells alone are not sufficient to support tissue repair. Mesenchymal stem cells (MSCs) are widely used in tissue engineering (Xing et al., 2019). Stem cells most commonly used to regenerate bone and tooth tissue are bone marrow mesenchymal stem cells (BMSCs) (Liu et al., 2020), adipose-derived stem cells (ADSCs) (Tobita and Mizuno, 2013), alveolar bone periosteum stem cells (PMSCs) (Wang YL et al., 2018),and dental mesenchymal stem cells (DSCs) (Gan et al., 2020), including periodontal ligament stem cells (PDLSCs) (Seo et al., 2004), dental** **pulp** **stem** **cells (DPSCs), dental follicle stem cells (DFSCs) (Han et al., 2010), stem cells from deciduous teeth (SHEDs) (Kunimatsu et al., 2018) and stem cells from the apical papilla (SCAPs) (Kang et al., 2019), and alveolar bone-derived mesenchymal stem cells (ABMSCs) (Matsubara et al., 2005) **Figure 2**. In addition, MSCs extracted from adipose tissue have strong differentiation and growth factor secretion potential, as well as strong bone formation ability (Alonso-Rodriguez et al., 2019). The strong proliferative and osteogenic differentiation abilities of PMSCs make them ideal materials for bone tissue regeneration (Wang YL et al., 2018). Xing et al. compared DPSCs, PDLSCs, and gingival MSCs (GMSCs), the three most readily available dental stem cells, for tissue regeneration (Xing et al., 2019). The authors found that there were significant differences in the number of passages and the ability of osteogenic differentiation among the three types of cells, suggesting that research should be directed to determine ways to promote the osteogenic differentiation of GMSCs and DPSCs, and to explore ways to increase the number of passages of PDLSCs. Xing et al. and Zhang et al. proposed that PDLSCs have good application prospects for bone regeneration (Zhang et al., 2018; Xing et al., 2019). Additionally, extracellular Vesicles (EVs) released by PDLSCs have osteogenic properties and promote bone regeneration (Gan et al., 2020). Moreover, bone marrow mesenchymal stem cell-derived small extracellular vesicles (BMSC-sEVs) may regulate osteoclast function and facilitate the migration, proliferation, and osteogenic differentiation of hPDLCs through the OPG-RANKL-RANK signaling pathway (Liu et al., 2021). Clinically, EVs are considered a potential medical strategy for cell-free regenerative therapy (Zheng et al., 2019). According to a previous study, exosomes secreted by GMSCs pretreated with TNF-α may also be a promising tool for the repair of inflammatory bone loss diseases (Nakao et al., 2021). SHEDs are likely to be a promising source of cell material for bone regeneration therapy, as demonstrated by the experimental results showing that SHEDs have higher proliferative activity and higher expression of BMP-2 compared to BMSCs and DPSCs (Kunimatsu et al., 2018). In addition, SHEDs promote blood vessel and bone formation through exosomes *via* the AMPK signaling pathway (Wu et al., 2019).

### 4.4 Innovative sources of MSCs

The MSCs used for periapical bone remodeling are not necessarily derived from normal body tissues. For example, human periapical cysts contain numerous MSCs and immature progenitor cells, with good osteogenic differentiation and proliferative activity (Tatullo et al., 2019). Wisdom teeth and lost baby teeth can be used effectively in a similar manner (**Figure 2**).

MSC culture conditioned medium (MSC-CM) is a mixture of hundreds of different cytokines, growth factors, proteins, and enzymes based on this mechanism. Lin et al. found that MSC-CM is safer and more effective than MSC transplantation for periodontal tissue regeneration (Lin et al., 2021). This is reflected in the fact that MSC-CM is expected to achieve the goal of not using autologous or allogeneic stem cells, and the concentration of the active ingredients in MSC-CM can be customized as required.

### 4.5 Combination researches of CGF and MSCs of osteogenic differentiation

Some progress has also been made in the cross-study of CGF and MSCs. It has been confirmed that CGF can act on the osteogenic differentiation of HDPSCs, BMSCs, and GMSCs (Chen X et al., 2019; Xu F et al., 2019; Rochira et al., 2020; Li Z et al., 2021). Interestingly, the promotion of osteogenic differentiation of hPDLCs by CGF does not appear to be affected by the inflammatory microenvironment (Li et al., 2019). CGF can directly induce osteogenic differentiation of hBMSC (Rochira et al., 2020) and regulate osteogenic differentiation of GMSCs (Chen X et al., 2019) and SCAPs (Hong et al., 2018) by upregulating osteogenic differentiation-related genes, such as RUNX2 and COL-I. It was also found that 10% CGF had the most significant promoting effect on MSC proliferation (Chen X et al., 2019). In addition, experimental studies revealed that human umbilical cord MSCs could upregulate the expression of COL-1, ALP, OCN, and RUNX2 and inhibit the expression of MMP1, while CGF could promote umbilical cord MSC-mediated tissue regeneration (Li W et al., 2021).

## 5 Conclusion and outlook

The pathological process of CAP is induced by predominantly bacterial pathogens, and studies have reported the contribution of viruses such as: EBV (Jakovljevic et al., 2020), varicella zoster virus (Heithersay and Chew, 2021) and other novel pathogens to the development of periapical inflammation.

The combination of pathogenic invasion and the response produced by the body expands the extent of the lesion. The body releases a large number of inflammatory-associated factors in the periapical inflammatory zone, leading to a range of inflammatory injuries including osteolysis. Changes in the expression levels of these inflammatory factors may be related to the DNA methylation status of the corresponding promoter region and the regulatory role of mRNA. In addition, the hypoxic environment created by periapical inflamed tissue can induce the production of reactive oxygen species and apoptosis of osteoblasts. The increase in reactive oxygen species stimulates RANKL to promote osteoclast differentiation for bone resorption.

The fact that periapical inflammatory tissue can recruit stem cells important for tissue repair to the lesioned area suggests that the body also has some self-healing function. However, this self-healing capacity is not sufficient to compensate for the inflammatory damage. Thus, CGFs and MSCs have become new research and clinical focuses because of their ability to promote tissue repair in lesions. In addition, EV and MSC-CM seem to provide a more effective and safe means for bone reconstruction, which deserves further exploration.

Before administering pro-repair substances, the first task is to remove pathogenic microorganisms from the root canal and periapical area. Due to the complex anatomy of the root canal system, conventional root canal treatment is unable to completely remove the pathogenic microorganisms, and the introduction of probiotic products offers a new way of solving this challenge.

## Authors contributions

XL, QW, LC and RX contributed to conception and design of the study. XL and QW contributed to the literature searching and analysis. XL, QW and RX organized and draw the figures. XL, QW and RX wrote the draft of the manuscript. All authors contributed to manuscript revision, read, and approved the submitted version.

## Conflict of interest

The authors declare that this paper was accomplished in the absence of any commercial or financial relationships that could be construed as a potential conflict of interest.

## Publisher’s note

All claims expressed in this article are solely those of the authors and do not necessarily represent those of their affiliated organizations, or those of the publisher, the editors and the reviewers. Any product that may be evaluated in this article, or claim that may be made by its manufacturer, is not guaranteed or endorsed by the publisher.
